# The Effectiveness of Digital Animation–Based Multistage Education for Patients With Atrial Fibrillation Catheter Ablation: Randomized Clinical Trial

**DOI:** 10.2196/65685

**Published:** 2025-03-11

**Authors:** Xiaoyu Shi, Yijun Wang, Yuhong Wang, Jun Wang, Chen Peng, Siyi Cheng, Lingpeng Song, Rui Li, Fuding Guo, Zeyan Li, Shoupeng Duan, Xiaomeng Yang, Liping Zhou, Hong Jiang, Lilei Yu

**Affiliations:** 1 Department of Cardiology Renmin Hospital of Wuhan University Wuhan China; 2 Institute of Molecular Medicine Renmin Hospital of Wuhan University Wuhan China; 3 Taikang Center for Life and Medical Sciences Wuhan University Wuhan China; 4 Hubei Key Laboratory of Autonomic Nervous System Modulation Wuhan University Wuhan China; 5 Hubei Key Laboratory of Cardiology Cardiovascular Research Institute Wuhan University Wuhan China; 6 Department of Cardiology The First Affiliated Hospital of Bengbu Medical University Bengbu China; 7 Department of Cardiology Yan'an Affiliated Hospital of Kunming Medical University Kunming China

**Keywords:** animation education, digital health care, atrial fibrillation, catheter ablation, video, mHealth, digital care, digital health, digital animation, randomized clinical trial, RCT, digital education, outpatient, AFCA, atrial fibrillation catheter ablation, therapeutic, cardiac arrhythmia, Asian, animations, comics

## Abstract

**Background:**

Digital education for outpatient patients with atrial fibrillation (AF) has gradually increased. However, research on digital education for patients undergoing atrial fibrillation catheter ablation (AFCA) is limited.

**Objective:**

This study aimed to develop a novel digital animation-based multistage education system and evaluate its quality-of-life benefits for patients with AFCA.

**Methods:**

This randomized controlled clinical trial included 208 patients with AF who underwent catheter ablation in the Department of Cardiology at Renmin Hospital of Wuhan University between January 2022 and August 2023. The patients were randomly assigned to the digital animation intervention (n=104) and standard treatment (n=104) groups. The primary outcome was the difference in the quality of life of patients with atrial fibrillation (AF-QoL-18) scores at 3 months. Secondary outcomes included differences in scores on the 5-item Medication Adherence Report Scale (MARS-5), Self-rating Anxiety Scale (SAS), and Self-Rating Depression Scale (SDS) at 3 months.

**Results:**

In the digital animation intervention group, the AF-QoL-18 score increased from 38.02 (SD 6.52) to 47.77 (SD 5.74), the MARS-5 score increased from 17.04 (SD 3.03) to 20.13 (SD 2.12), the SAS score decreased from 52.82 (SD 8.08) to 45.39 (SD 6.13), and the SDS score decreased from 54.12 (SD 6.13) to 45.47 (SD 5.94), 3 months post discharge from the hospital. In the conventional treatment group, the AF-QoL-18 score increased from 36.97 (SD 7.00) to 45.31 (SD 5.71), the MARS-5 score increased from 17.14 (SD 3.01) to 18.47 (SD 2.79), the SAS score decreased from 51.83 (SD 7.74) to 47.31 (SD 5.87), and the SDS score decreased from 52.78 (SD 5.21) to 45.37 (SD 6.18). The mean difference in AF-QoL-18 score change between the 2 groups was 1.41 (95% CI 2.42-0.40, *P*=.006) at 3 months. The mean difference in MARS-5 score change was 1.76 (95% CI 2.42-1.10, *P*<.001). The mean difference in SAS score was –2.91 (95% CI –3.88 to –1.95, *P*<.001). Additionally, the mean difference in SDS score was –1.23 (95% CI –0.02 to –2.44, *P*=.047).

**Conclusions:**

Our study introduces a novel digital animation educational approach that provides multidimensional, easily understandable, and multistage education for patients with AF undergoing catheter ablation. This educational model effectively improves postoperative anxiety, depression, medication adherence, and quality of life in patients at 3 months post discharge.

**Trial Registration:**

Chinese Clinical Trial Registry ChiCTR2400081673; https://www.chictr.org.cn/showproj.html?proj=201059

## Introduction

Atrial fibrillation (AF) is the most common cardiac arrhythmia and significantly increases the risk of mortality, stroke, heart failure, and dementia, thereby severely impacting patient quality of life (QoL) [1]. It is estimated that by 2050, at least 72 million Asian individuals will be diagnosed with AF [1,2]. The primary goals to treat AF include symptom improvement, heart rate and rhythm control, and stroke risk reduction [3,4]. Currently, catheter ablation has progressively emerged as the primary therapeutic approach for rhythm control in AF, owing to its potential to mitigate AF episodes and enhance patients’ QoL [3,5]. However, the efficacy of catheter ablation in reducing AF remains uncertain with regard to its impact on patients’ QoL.

Studies have indicated that negative emotions, alcohol consumption, smoking, obesity, and sleep disorders, among other high-risk factors, can expedite AF progression and prognosis, potentially directly impacting the efficacy of atrial fibrillation catheter ablation (AFCA) and postoperative recovery [6]. Additionally, anticoagulation therapy significantly reduces the stroke risk in patients with AF and is emerging as a crucial factor affecting treatment efficacy [7,8]. Therefore, alongside enhancing the safety and QoL benefits of AFCA, strategies focusing on risk factor management and medication adherence to improve ablation outcomes are essential in patients with AF [9,10].

Popular science interventions, such as animations and comics, are a noninvasive, entertaining, emerging approach to disease intervention used to ameliorate patient emotions, sleep, lifestyle, and disease prognosis [11-13]. Patients can vividly comprehend various aspects of AF, including etiology, pathology, surgical procedures, and complications, using vivid and engaging animation content, thereby enhancing their interest and engagement in health management. A recent study suggested that using animation education can enhance patients’ understanding of AF and increase their satisfaction with clinical care [14].

With the advancement of internet technology, an increasing number of patients obtain information regarding AF via web-based videos. However, most videos vary in the direction and quality of their focus and offer limited benefits to patients undergoing AFCA [15]. Hence, new and more accessible digital animations are needed, and patients should be provided with digital education during the hospitalization and postdischarge phases of catheter ablation to facilitate management at different stages of AF. In this prospective, randomized, controlled clinical trial, we aimed to evaluate the benefits of multistage education based on digital animation for patients with AF undergoing catheter ablation.

## Methods

### Study Design

The trial protocol followed the guidelines outlined in the CONSORT-EHEALTH (Consolidated Standards of Reporting Trials of Electronic and Mobile Health Applications and Online Telehealth) checklist (Multimedia Appendix 1) for clinical trial reporting. Written informed consent was obtained from all patients. The complete protocol and relevant materials are presented in Multimedia Appendix 2.

### Study Population

This randomized controlled trial enrolled 208 patients with AF who underwent AFCA in the Department of Cardiology at Renmin Hospital of Wuhan University between January 2022 and August 2023. The primary inclusion criteria were as follows: (1) aged between 18 and 90 years, (2) confirmed diagnosis of AF via surface electrocardiogram (ECG) or ambulatory ECG examination, and (3) patients with AF who received AFCA for the first time and were successfully treated during their stay at Renmin Hospital of Wuhan University. The procedures for both groups were conducted by the same AFCA team, ensuring consistency in the ablation techniques used. All the AF diagnosis and treatment criteria strictly adhered to the AF management [16]. Participants failing to meet the study criteria, as determined by the clinical investigator, were excluded if they scored below the designated threshold on the Mini-Mental State Examination or had a medical history of percutaneous coronary intervention or coronary artery bypass grafting. The detailed inclusion and exclusion criteria are provided in Multimedia Appendix 2.

### Randomization and Masking

A web-based randomization tool was used to allocate 208 patients undergoing radiofrequency ablation to either the digital animation intervention group or the standard treatment group, with equal probability. Stratification based on gender and age (>60 or ≤60 years) was conducted to ensure a balance in baseline characteristics between the 2 groups. The blinding of participants was unfeasible given the inherent nature of the intervention.

### Study Procedures

Participants were assessed at baseline (upon admission) and 3 months post discharge. During the recruitment process, baseline datasets containing sociodemographic characteristics and medical histories were obtained from the digital animation intervention and control groups. Standardized questionnaire surveys were conducted, including the quality of life of patients with atrial fibrillation (AF-QoL-18) [17,18], Self-rating Anxiety Scale (SAS) [19], Self-Rating Depression Scale (SDS) [20] and 5-item Medication Adherence Report Scale (MARS-5) [21,22]. Information regarding at-home medication use was collected from the patients in both groups at the time of discharge. Patients completed the standardized questionnaires at the 3-month follow-up. Patient characteristics and outcomes were collected by reviewing medical records of trained clinical physicians and participant self-reports. Multimedia Appendix 2 contains details of data acquisition in the study protocol.

### Study Interventions

**Figure 1:**
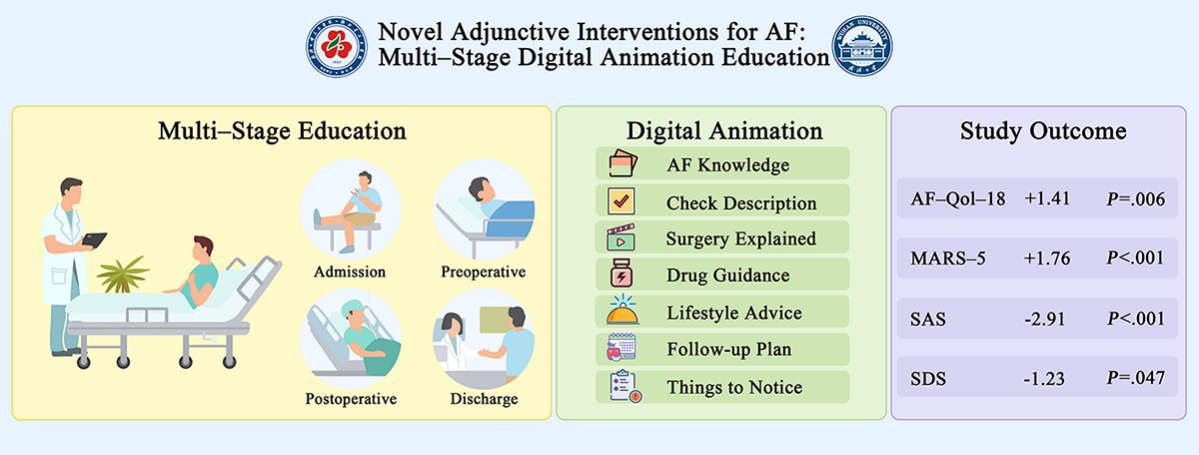
This study presents a visual summary of a digital animation-based educational framework for AF management, structured into 4 distinct phases. The first phase, admission, involves the initial education of patients about AF. The second phase, preoperative, employs digital animation to elucidate the surgical procedure. The third phase, postoperative, offers comprehensive guidance on medication and lifestyle modifications. The final phase, discharge, focuses on developing a structured follow-up plan. The digital animation comprehensively covers essential topics such as AF Knowledge, Check Description, Surgery Explained, Drug Guidance, Lifestyle Advice, Follow-Up Plan, and Things to Notice. The outcomes of the study demonstrate significant improvements, including AF-QoL-18 (*P*=.006), MARS-5 (*P*<.001), SAS (*P*<.001), and SDS (*P*=.047). AF: atrial fibrillation; AF-Qol-18: atrial fibrillation effect on quality-of-life scale; MARS-5: 5-item Medication Adherence Rating Scale; SAS: Self-rating Anxiety Scale; SDS: Self-Rating Depression Scale.

Based on the recommendations of the guidelines for treating AF, doctors provided all patients with standardized treatment and knowledge regarding AFCA [16]. In the digital animation intervention group, doctors conducted regular knowledge lectures to patients and used customized digital animations during treatment to educate them, ensuring that patients effectively received relevant information regarding AF treatment at appropriate stages. Figure 1 provides a visual summary of digital animation education for AF.

In the first phase, physicians provided standardized iPad devices (Apple) to facilitate playback and guided patients in the digital animation intervention group to watch 4 AF-related animations during admission, preoperative, postoperative, and discharge periods. These animations encompassed various aspects as follows: (1) imparting knowledge on cardiac anatomy, mechanisms underlying AF occurrence, and associated risk factors, as well as explaining the necessary examinations before catheter ablation and their significance; (2) discussing the purpose, process, and precautions of catheter ablation surgery; (3) outlining postoperative care requirements during hospital stay following catheter ablation; (4) emphasizing medication adherence upon discharge along with lifestyle recommendations and plans for regular follow-up visits. Furthermore, physicians addressed any queries raised by patients after viewing the animations to enhance comprehension. Patients in the control group solely received routine preoperative discussions without access to digitally animated educational videos at different stages. All patients completed a comprehensive study scale upon admission.

In the second phase, patients in the intervention and control groups were followed up 3 months after discharge by telephone to complete the study questionnaire. Multimedia Appendix 2 provides a detailed description of the intervention activities and their implementations.

All animation contents were directed and produced under the guidance of multiple cardiologists with extensive expertise. This ensured that the animations included important information about the diagnosis and treatment of diseases, surgeries, and crucial aspects of home-based rehabilitation, including improvements in sleep, emotional care, dietary adjustments, medication adherence, and activity recommendations. To minimize barriers to health education dissemination and language obstacles, animations were created based on elementary literacy levels, with audio and subtitles provided in standard Mandarin Chinese. However, each animation was strictly controlled for <5 min. Additionally, regular quality control and feedback mechanisms were implemented to ensure intervention effectiveness.

Regardless of the study group, all patients received standard hospital education from health care professionals and could discuss any questions or concerns regarding AF treatment with their primary physicians. All patients underwent AFCA and were followed for 3 months post discharge.

### Measurements

BMI, calculated as weight (kg) divided by height (m^2^), was obtained from the self-reported height and weight.

Measures related to psychometrics and psychological aspects, including the AF-QoL-18, SAS, SDS, and MARS-5, were used. The AF-QoL-18 was used to assess QoL in patients with AF. The SAS was used to assess individual levels of anxiety. The SDS was used to measure depression severity. The MARS-5 was used to evaluate patient medication adherence. The details of the QoL scores and related quality controls are listed in Multimedia Appendix 2.

### Outcomes

Clinical outcome measurements were obtained at the 3-month follow-up. The primary outcome was the difference in AF-QoL-18 scores at 3 months, while the secondary outcomes included differences in MARS-5, SAS, and SDS scores. The definitions of the clinical outcomes and details regarding quality control are presented in Multimedia Appendix 2.

### Ethical Considerations

This randomized controlled clinical study was approved by the Research Ethics Committee of Renmin Hospital of Wuhan University (approval number WDRY2021-K161) and registered with the Chinese Clinical Trial Registry (registration number ChiCTR2400081673). The trial was conducted in accordance with good clinical practice guidelines, the Declaration of Helsinki, and all relevant laws and regulations in China.

Written informed consent was obtained from all participants prior to enrollment. The consent forms detailed the study objectives, procedures, potential risks, and benefits, as well as data use policies. The informed consent documents have been uploaded as part of this submission (Multimedia Appendix 2).

No identifiable images of participants are included in the manuscript or supplementary materials. Identifiable information was removed from the datasets prior to analysis. All study data were anonymized and deidentified to protect participant privacy. All consent forms explicitly authorized the use of anonymized data for research publication.

The study involved only standard clinical care for AFCA and a digital animation-based multistage education program, which is categorized as routine and noninvasive patient education. In accordance with local regulations for low-risk clinical trials and the ethical approval guidelines of Renmin Hospital of Wuhan University, no financial compensation was provided to participants due to the minimal-risk nature of the study.

### Statistical Analysis

The sample size was estimated by comparing 2 independent proportions with a type I error rate (α) set of 2.5%, an intracluster correlation coefficient of 0.01, and an anticipated retention rate of 80%. A significant absolute difference of 20% in clinical outcomes was detected between the intervention and standard treatment groups, with a statistical power of 90% at a follow-up period of 3 months. Based on our calculations, a minimum sample size of 80 assessable patients per group was required to achieve the desired statistical power.

Statistical analyses were conducted using SPSS (version 23.0; IBM Corp) and R (version 4.2.2; R Foundation for Statistical Computing) software. Data with normal distribution are presented as mean (SD). The 2 groups were compared using the independent sample *t* test. Data with nonnormal distributions are presented as medians and interquartile ranges. The Mann-Whitney *U* test was used to analyze the differences between the groups. Categorical data are expressed as numbers and percentages and compared using the chi-square test or Fisher exact test, as appropriate. To assess the effectiveness of the intervention, the differences between the 2 groups were calculated by subtracting the change from baseline to 3 months in the control group from that in the intervention group. Then, an independent *t* test was used to compare differences between groups before and after the intervention. A *P*<.05 was considered statistically significant.

## Results

**Figure 2:**
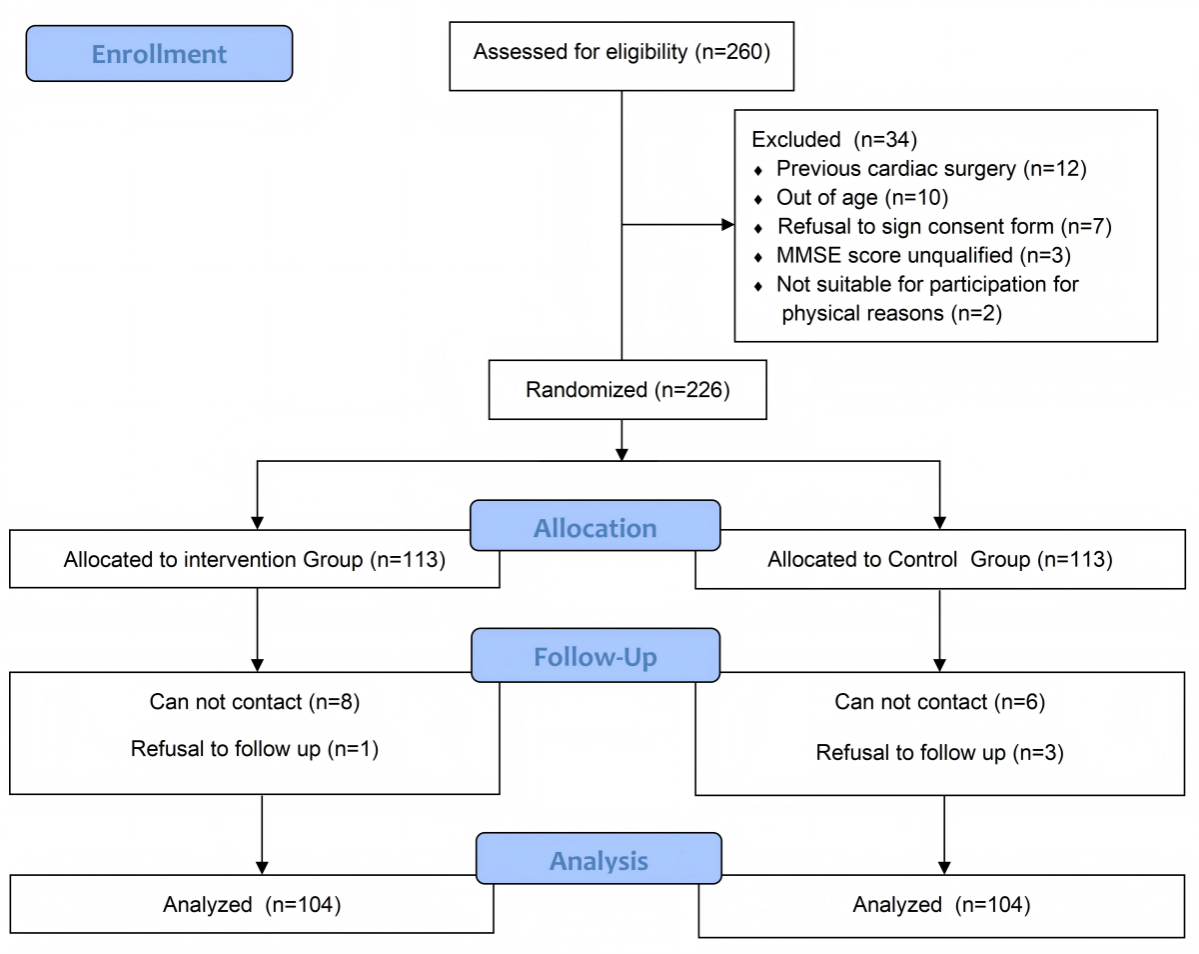
Screening, randomization, and follow-up flowchart. Our study recruited patients diagnosed with atrial fibrillation who were scheduled for catheter ablation at the Department of Cardiology, Renmin Hospital of Wuhan University, between January 2022 and August 2023. Following a rigorous screening process, eligible participants were randomly assigned to either the digital animation intervention, which received digital animation-based education, or the standard treatment groups, which received standard care. The follow-up period was 3 months. A total of 208 patients with complete data were included in the final analysis. MMSE: Mini-Mental State Examination.

**Figure 3:**
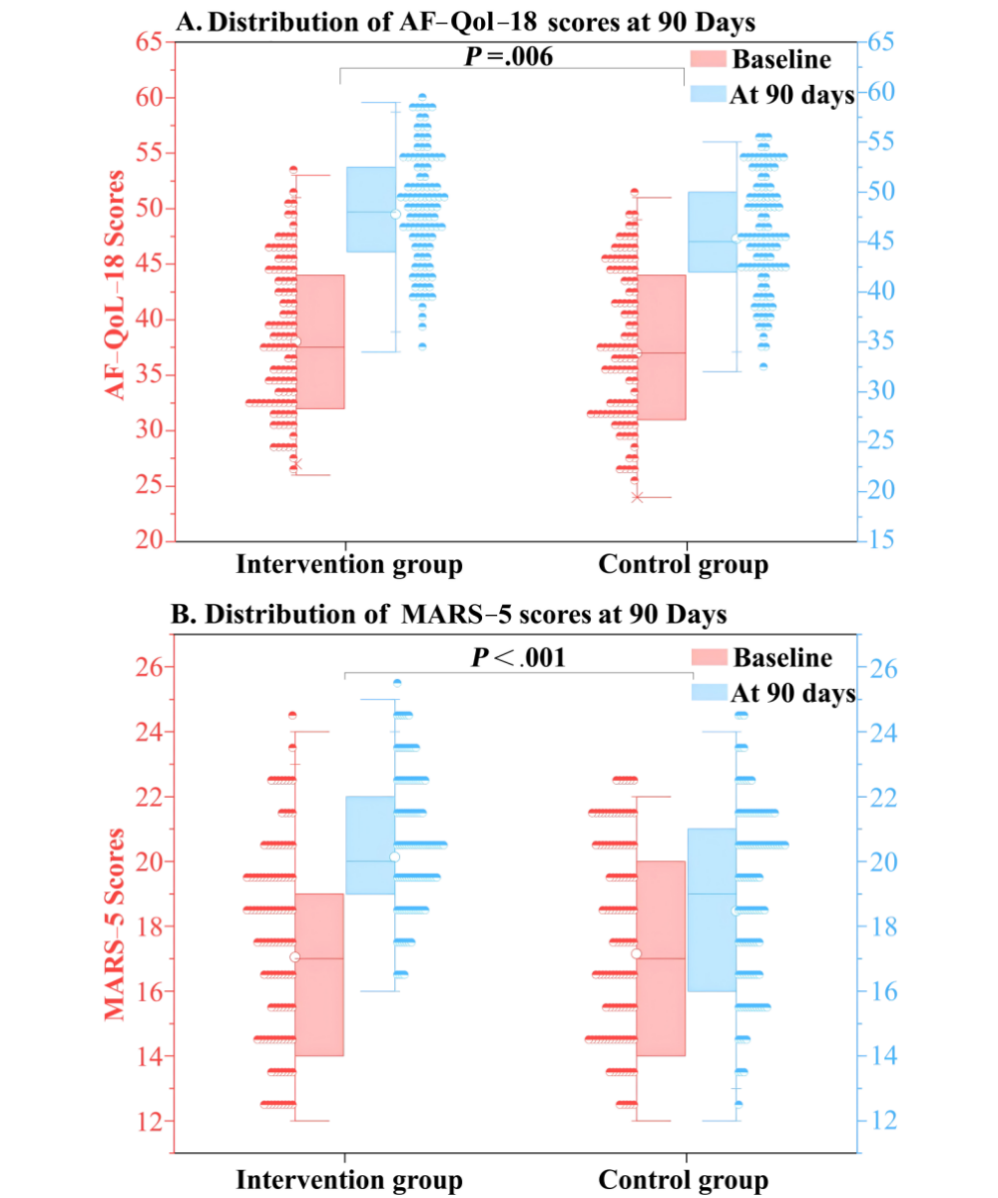
Comparison of AF-QoL-18 and MARS-5 scores between the digital animation intervention and standard treatment groups in patients with atrial fibrillation at baseline and 3-month follow-up. AF-Qol-18: atrial fibrillation effect on quality-of-life Scale; MARS-5: 5-item Medication Adherence Rating Scale.

**Figure 4:**
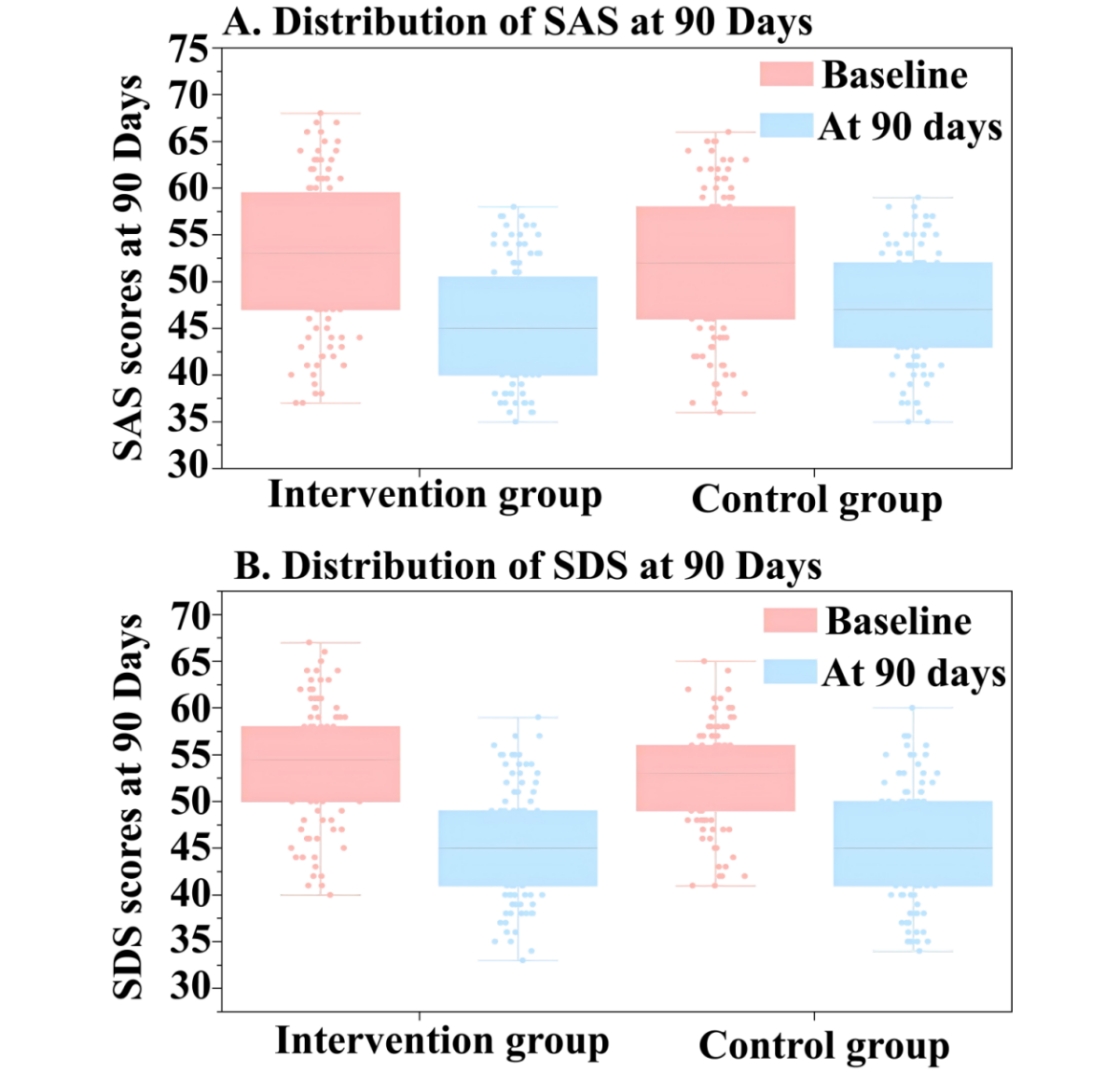
Comparison of SAS and SDS scores between the digital animation intervention and standard treatment groups in patients with atrial fibrillation at baseline and at 3 months follow-up. SAS: Self-rating Anxiety Scale; SDS: Self-Rating Depression Scale.

Figure 2 illustrates participant recruitment and retention. Among 208 patients undergoing catheter ablation (average age of 60.9 years), 68 (32.7%) were female and 140 (67.3%) were male. At baseline, the participants were randomly assigned to the digital animation intervention (n=104) and standard treatment (n=104) groups, both of which had similar characteristics. Table 1 presents the baseline characteristics of the digital animation intervention and the standard treatment groups. Table S1 in Multimedia Appendix 3 reveals significant statistical improvements in the AF-QoL-18, MARS-5, SAS, and SDS scores (all *P*<.001) at 3 months compared with baseline. In the digital animation intervention group, the AF-QoL-18 score increased from 38.02 (SD 6.52) to 47.77 (SD 5.74; *P*<.001; Figure 3A), the MARS-5 score increased from 17.04 (SD 3.03) to 20.13 (SD 2.12; *P*<.001; Figure 3B), the SAS score decreased from 52.82 (SD 8.08) to 45.39 (SD 6.13; *P*<.001; Figure 4A), and the SDS score decreased from 54.12 (SD 6.13) to 45.47 (SD 5.94; *P*<.001; Figure 4B). In the conventional treatment group, the AF-QoL-18 score increased from 36.97 (SD 7.00) to 45.31 (SD 5.71; *P*<.001), the MARS-5 score increased from 17.14 (SD 3.01) to 18.47 (SD 2.79; *P*<.001), the SAS score decreased from 51.83 (SD 7.74) to 47.31 (SD 5.87; *P*<.001), and the SDS score decreased from 52.78 (SD 5.21) to 45.37 (SD 6.18; *P*<.001; Table S1 in Multimedia Appendix 3).

The AF-QoL-18 (*P*=.002), MARS-5 (*P*<.001), and SAS (*P*=.02) scores of the digital animation intervention and conventional treatment groups were significantly different at 3 months. However, the SDS score (*P*=.90) did not exhibit a statistically significant difference (Table S2 in Multimedia Appendix 3). Additionally, the mean difference in AF-QoL-18 score change between the 2 groups was 1.41 (95% CI 2.42-0.40) at 3 months (*P*=.006; Table S3 in Multimedia Appendix 3). The mean difference in MARS-5 score change was 1.76 (95% CI 2.42-1.10, *P*<.001; Table S3 in Multimedia Appendix 3). The mean difference in SAS score was –2.91 (95% CI –3.88 to –1.95, *P*<.001; Table S3 in Multimedia Appendix 3). Furthermore, the mean difference in SDS score was –1.23 (95% CI –0.02 to –2.44), indicating an estimated difference between the digital animation intervention and conventional treatment groups at 3 months (*P*=.047; Table S3 in Multimedia Appendix 3).

At the 3-month follow-up, all patients underwent 12-lead ECGs and 24-hour ambulatory ECG monitoring to assess rhythm control. Our findings indicated that 9 patients in the digital animation intervention group experienced atrial arrhythmic events, including AF, atrial flutter, and atrial tachycardia, compared with 13 patients in the control group. However, the difference in the recurrence rates of these arrhythmic events between the 2 groups was not statistically significant (*P*=.50; Table S4 in Multimedia Appendix 3). Additionally, the mean heart rate was 70.08 bpm in the digital animation intervention group and 70.85 bpm in the control group. The difference in mean heart rate between the 2 groups was also not statistically significant (*P*=.25; Table S4 in Multimedia Appendix 3). Neither group experienced serious cardiovascular events, and no adverse reactions related to the medications were reported in either group.

**Table 1:** Demographic and baseline clinical characteristics at baseline.

Characteristic		Intervention group (n=104)	Control group (n=104)
Male, n (%)		70 (67.3)	70 (67.3)
Age (years), mean (SD)		61.18 (10.90)	60.70 (11.33)
BMI, (kg/m^2^), mean (SD)		24.61 (3.07)	23.96 (3.20)
**Marital status, n (%)**			
	Married	97 (93.3)	98 (94.2)
	Single	2 (1.9)	4 (3.8)
	Widowed	5 (4.8)	2 (1.9)
**Smoking status, n (%)**			
	Smoker	17 (16.3)	16 (15.4)
	Former smoker	18 (17.3)	18 (17.3)
	Nonsmoker	69 (66.3)	70 (67.3)
**Drinking status, n (%)**			
	Drinker	16 (15.4)	27 (26.0)
	Former drinker	8 (7.7)	7 (6.7)
	Nondrinker	80 (76.9)	70 (67.3)
**Education, n (%)**			
	Elementary school	25 (24.0)	23 (22.1)
	Middle/high school	56 (53.8)	55 (52.9)
	University degree	23 (22.1)	26 (25.0)
Coronary artery disease, n (%)		26 (25.0)	25 (24.0)
Hypertension, n (%)		44 (42.3)	53 (51.0)
Diabetes, n (%)		21 (20.2)	20 (19.2)
Admission heart rate, mean (SD)		80.81 (16.06)	81.68 (18.21)
Admission systolic blood pressure (mm Hg), mean (SD)		129.22 (18.44)	125.59 (17.76)
Admission diastolic blood pressure (mm Hg), mean (SD)		76.82 (11.88)	76.58 (10.51)
Hospitalization duration (day), mean (SD)		10.97 (4.04)	11.28 (3.94)
**Type of atrial fibrillation, n (%)**			
	Paroxysmal atrial fibrillation	69 (66.3)	65 (62.5)
	Persistent atrial fibrillation	35 (33.7)	39 (37.5)
**Medication at discharge, n (%)**			
	Amiodarone	81 (77.9)	80 (76.9)
	Warfarin	39 (37.5)	37 (35.6)
	Rivaroxaban	55 (52.9)	58 (55.8)
	Atorvastatin	58 (55.8)	60 (57.7)
	β-blockers	30 (28.8)	38 (36.5)
	Angiotensin-converting enzyme inhibitor or angiotensin II receptor blocker	30 (28.8)	34 (32.7)
	Calcium channel blocker	14 (13.5)	16 (15.4)

## Discussion

### Principal Findings

This study demonstrates that multistage education based on digital animation improves the QoL and medication adherence of patients with AF and helps them improve their emotional well-being compared with conventional treatment. Digital animation-based multistage education serves as an adjunctive tool, providing visual cardiovascular disease knowledge and explaining necessary diagnostic procedures, the AFCA process, and treatment-related considerations. It also emphasizes the importance of medication adherence during rehabilitation and provides recommendations for lifestyle and behavioral changes, thereby amplifying the QoL benefits of AFCA.

### Comparison With Prior Work

In clinical practice, AFCA has emerged as a critical interventional strategy for rhythm control in patients with AF [5]. This procedure specifically targets and ablates ectopic triggering foci and abnormal electrical conduction pathways that are responsible for the initiation and perpetuation of AF [5]. By effectively disrupting these arrhythmogenic substrates, AFCA aims to restore and maintain normal sinus rhythm, thereby improving patient outcomes [5]. However, some patients experience suboptimal long-term outcomes [23]. This phenomenon may be attributed to a lack of understanding of AF, failure to eliminate risk factors, or poor medication adherence, directly impacting the effectiveness of AFCA and postoperative recovery [24-26]. Patient education regarding AF is crucial for reducing these adverse effects. Digital animation, a form of patient education, offers a more engaging and informative approach due to its audiovisual characteristics. This mode of patient education is more effective than traditional methods, such as visual brochures or one-on-one verbal conversations [27-29]. Recently, a randomized clinical trial evaluated the impact of video education created by doctors on patients with AF [14]. The results indicated that video education effectively improved AF knowledge and nursing satisfaction among outpatients with AF.

Previous animations used to educate patients with AF primarily focused on AF risk factors, symptoms, complications, and pharmacological and nonpharmacological treatments. However, knowledge regarding the procedural aspects of patients undergoing ablation surgery [30,31]. Unfamiliarity with the surgical environment and lack of understanding of the surgical procedure are important factors contributing to preoperative anxiety among patients [32,33]. This results in preoperative sleep disorders and adversely impacts postoperative efficacy and recovery [34]. Explaining the purpose, process, and precautions of surgery can help alleviate preoperative anxiety during the waiting period. A randomized controlled study demonstrated that combining audiovisual methods to educate patients about preoperative preparation, intraoperative procedures, and postoperative intensive care unit admission effectively reduced preoperative anxiety among patients undergoing coronary artery bypass grafting surgery [35]. Additionally, research using virtual reality devices to provide patients with a first-person perspective of the operating room and surgical procedures found a reduction in preoperative negative emotions and increased postoperative satisfaction [33]. These results indicate that educating patients about surgical procedures can improve their emotional state and benefit them in various aspects, including mental well-being and QoL, during the perioperative period.

In this study, targeted digital animation was designed for patients with AF undergoing AFCA. Compared with traditional audiovisual educational materials for patients with AF, this animation included additional information regarding the purpose, surgical process, intraoperative aspects, and postoperative considerations of catheter ablation. These contents helped reduce patient anxiety during hospitalization, deepen patient understanding of AF, promote changes in unhealthy lifestyles, and improve patient medication adherence, ultimately contributing to QoL enhancement in patients with AF [36-38].

Numerous people have recognized the advantages of video education over traditional educational methods, and numerous studies have produced professional educational videos. However, in clinical practice, most patient education was conducted as a single event during outpatient or inpatient visits [30,31]. Regular and repeated education helps improve patient knowledge of the disease over time. Studies have demonstrated that the relevant knowledge acquired by patients with AF significantly declines 2 weeks after receiving education, while multiple educational sessions help enhance patients’ awareness of the disease [14,39,40]. Compared with previous research, this experiment made a preliminary attempt by dividing surgery-related educational materials into preoperative and postoperative stages. Moreover, videos were sent to the patients for reinforced education after discharge. Instead of completing all educational content in a single session, this experiment used a multistage approach, effectively reducing the time required for patients to receive education, with each stage lasting <5 minutes. Patients with AF are mostly older people, particularly those with lower cognitive function, and receiving all educational information in one session can be challenging [41]. The short duration and highly focused nature of these videos ensured that the patients maximized their attention and minimized the opportunity for attentional decline. Furthermore, this staged intervention approach helped patients retain information from different stages of education to some extent, thereby improving medication adherence after discharge.

### Limitations

This study has several limitations to consider when interpreting the results. First, the sample size was relatively small (208 patients), and the study was conducted in a single hospital. Therefore, the generalizability of the results to larger populations or different health care settings is limited and requires further investigation. Second, the follow-up period was only 3 months, which may not capture the long-term educational effects and stability of the digital animation intervention. A longer follow-up may provide a more comprehensive understanding of the sustained impact of educational interventions. Third, the perioperative 3-month period, clinically referred to as the “blanking period,” is characterized by heightened vulnerability to diverse arrhythmic phenomena. This interval poses significant risks for patients, as electrophysiological instabilities can exacerbate underlying symptoms and compromise daily functional status. Therefore, meticulous monitoring is essential during this phase to optimize risk stratification and guide therapeutic decision-making. Fourth, the use of self-reported measures for medication adherence, anxiety, and depression may introduce response bias, as participants might overestimate their adherence or underreport symptoms due to social desirability. Fifth, while the digital animation system was designed with user-friendliness in mind, variations in individual patient engagement and technological proficiency may have exerted an influence on the efficacy of the intervention. It is important to acknowledge these limitations as they may impact the overall interpretation and generalizability of the findings of the study. Future research with larger and more diverse samples, longer follow-up periods, and objective outcome measures may help strengthen the evidence regarding the educational effectiveness of digital animation for patients with AF undergoing catheter ablation.

### Conclusions

Our study introduced a novel approach to digital animation education, providing multidimensional and highly accessible multistage education for patients with AF undergoing catheter ablation. This educational model has demonstrated effectiveness in improving postoperative anxiety, depression, medication adherence, and QoL in patients at the 3-month postdischarge follow-up.

## Appendix

Multimedia Appendix 1 CONSORT-eHEALTH checklist (V 1.6.1).

Multimedia Appendix 2 Trial Protocol.

Multimedia Appendix 3 Data Analysis.

## Abbreviations

AF: atrial fibrillation
AFCA: atrial fibrillation catheter ablation
AF-QoL-18: quality of life of patients with atrial fibrillation
CONSORT-EHEALTH: Consolidated Standards of Reporting Trials of Electronic and Mobile Health Applications and Online Telehealth
ECG: electrocardiogram
MARS-5: 5-item Medication Adherence Report Scale
QoL: quality of life
SAS: Self-rating Anxiety Scale
SDS: Self-Rating Depression Scale

## Data Availability

The datasets generated or analyzed during this study are available from the corresponding author on reasonable request.
