# Supplementary material for: The Effectiveness of Digital Animation–Based Multistage Education for Patients With Atrial Fibrillation Catheter Ablation: Randomized Clinical Trial
Source: J Med Internet Res. 2025 Mar 11;27:e65685. doi: 10.2196/65685 (PMC11937711; doi:10.2196/65685)
Supplement: Multimedia Appendix 2 [file jmir_v27i1e65685_app2.pdf]

Efficacy of a Digital Animation-based Multistage Education in Patients with Atrial Fibrillation Catheter ablation :Trial protocol and statistical analysis plan

Study title: Efficacy of a Digital Animation-based Multistage Education in Patients with Atrial Fibrillation Catheter ablation : A Randomized Clinical Trial.

Brief Title: Benefits of education targeting Atrial Fibrillation

Principal Investigator： Lilei Yu, MD, PhD, FESC, FACC

Study Center: Department of Cardiology, Renmin hospital of Wuhan University

Address for correspondence: No.238 Jiefang Road, Wuhan City, Hubei Province 430060, P.R. China

Contact Details: **E-mail: lileiyu@whu.edu.cn**

Study Number: No. WDRY2021-K161

Trial Registration: No. ChiCTR2400081673

Funding Support: This work was supported by grants from the National Natural Science Foundation of China(82270532, 82241057), Foundation for Innovative Research Groups of Natural Science Foundation of Hubei Province, China(2021CFA010), Chutian Talent Program Science and Technology Innovation Team Project, Central University Basic Scientific Research Business Expenses Special Funds(2042024kf0041, 2042022kf1211), Taikang Life Medicine Central PI Program of Wuhan University and Talent Program of Renmin Hospital, Wuhan University(JCRCYG-2022-001).

Original Protocol: Version 1.0 August 18, 2021

Introduction

Atrial Fibrillation (AF) is the most common arrhythmia, and its significant increase in prevalence has led to heightened risks of heart failure and stroke within the population[1]. It is projected that by 2050, at least 72 million individuals in Asia will be diagnosed with AF, with approximately 3 million potentially experiencing AF-related strokes[2]. Furthermore, AF imposes a considerable psychosocial burden on patients, including anxiety and depression, which significantly diminishes their quality of life[3, 4]. The primary objectives of AF treatment include symptom relief, heart rate and rhythm control, and stroke risk reduction. AF catheter ablation (AFCA) has emerged as the most prevalent cardiac ablation procedure globally and is a critical strategy for rhythm control. However, the long-term prognosis remains unsatisfactory for some patients[5]. Therefore, alongside enhancing the safety and efficacy of AFCA, strategies that improve ablation outcomes through lifestyle interventions and risk factor management are also regarded as vital goals in the management of AF patients.

Educational interventions utilizing videos and comics represent a non-invasive and engaging emerging approach to disease management, aimed at improving patients' emotional well-being, sleep quality, and lifestyle, potentially influencing disease prognosis. Research indicates that animated interventions for patients with acute coronary syndrome can enhance disease awareness, medication beliefs, and prognosis by educating them about their condition and the necessity of procedures or referrals[6]. A randomized controlled trial by Anna et al. demonstrated that among 135 patients requiring coronary angiography, those in the comic illustration group exhibited significantly improved understanding of the procedure and satisfaction, alongside reduced anxiety levels[7].

Currently, there is limited research focusing on the impact of video interventions on surgical satisfaction, emotional state, and sleep quality in patients undergoing AFCA. This study proposes an innovative strategy for the treatment of AF through video interventions and underscores the necessity of addressing this critical issue in the future management of AF patients. Given the substantial economic burden AF places on the healthcare system in our country, this research aims to further explore

the effects of combined catheter ablation and video interventions on patients' anxiety, depression, medication adherence, and quality of life. We anticipate that this study will advance AF treatment strategies, provide robust support for clinicians, and facilitate patient recovery.

Study Rationale

We used digital animation to educate patients. Digital animation has the characteristics of humor, easy to understand, interesting, and patients are more likely to accept, good adherence and easy to understand. Science education is different from before video, we provide all the digital animation designed by wuhan university people's hospital of professional medical personnel, can fully guarantee the quality of the video content. In addition, we produced a series of popular science videos, including the pathogenesis of AF, surgical methods of AFCA, preoperative preparation and examination of AFCA, postoperative precautions of AFCA, and suggestions for out-of-hospital lifestyle, covering all aspects of patients' onset, treatment and lifestyle. In multi-phase digital animation education mode, we adopted many times means of intervention, since found in patients with disease in hospitalized patients, treatment, discharge, and other key point is to intervene.

Multi-phase digital animation education process accompanied by paramedics offline to complete all the way, in the process of physicians were also responsible for solve doubts, maximize helps people understand and learn animation. The time node of multi-period intervention is the key time point for patients to be confused about the disease, treatment and lifestyle, which exactly needs the communication of doctors to help patients understand, reduce the negative emotions of patients, and improve the degree of medical cooperation and satisfaction of patients. Multi-stage digital animation education mode can help medical staff to complete doctor-patient communication efficiently, conveniently and visually, which is easy for patients to accept and understand. This model to better complete the doctor-patient communication is an important link, can effectively reduce the doctor-patient contradiction. At the same time, it can effectively improve the cognition and

compliance of patients, and improve the satisfaction of patients. It is worth noting that multi-stage digital animation education is only a mode to assist doctors to better complete the doctor-patient communication, and can never replace doctors. Doctors are the main carrier of doctor-patient communication and medical process.

Now, most current medical decision-making focuses only on the patients with disease occurrence, development, ignored the patients related issues. Patients in the hospital during the period of emotions and satisfaction are still part of the treatment process should be the doctor should not be neglected. More time digital animation education model in helping patients at the same time, increase the doctor's working efficiency, reduce the workload. It can improve the participation of patients in the process of disease diagnosis and treatment, help patients better complete medical decision-making, and become the first responsible person for their own health.

Study Purpose

Primary Objective: To assess the improvement in quality of life among patients with AFCA three months post-discharge.

Secondary Objectives: To evaluate the changes in anxiety, depression, and medication adherence among patients with AFCA three months after discharge.

Study Design

The study is designed to be a randomized, controlled trial conducted to test the therapeutic benefit of multi-stage digital animation education with AF.

Our study was conducted at the Department of Cardiology, Renmin Hospital of Wuhan University, a tertiary academic medical center renowned for its extensive clinical experience in cardiovascular interventional procedures. The hospital's AFCA team specializes in managing complex arrhythmia cases, ensuring comprehensive and high-quality care for patients undergoing AFCA. Potential participants were identified from the hospital’s AFCA patient registry, which encompasses all patients with a confirmed AF diagnosis verified by 12-lead ECG or Holter monitoring. Our research team meticulously reviewed electronic health records (EHR) to screen for eligibility based on predefined criteria. Identified patients were approached during

pre-admission consultations or via telephone prior to admission to provide detailed information about the study.

During recruitment contacts, a standardized protocol was strictly followed to ensure that participants were fully informed about the study’ s purpose, procedures, potential risks, and benefits. Our research team provided a thorough explanation of the intervention (digital animation-based education versus standard care) and addressed any questions. Participants who received this information were invited to sign the informed consent form upon admission. Only those who voluntarily consented were enrolled in the study. Once informed consent was obtained, baseline data were collected to confirm eligibility, including medical history (AF subtype, comorbidities, prior treatments), demographics (age, gender, ethnicity), vital signs (blood pressure, heart rate), and diagnostic results (echocardiogram, Holter monitor). This ensured that all participants met the eligibility criteria before proceeding with randomization.

The recruitment period extended from January 3, 2022, to August 18, 2023. During this time, continuous monitoring for any adverse events was conducted throughout the follow-up period. Both recruitment and follow-up adhered strictly to the ethical guidelines approved by the Institutional Review Board of Renmin Hospital of Wuhan University.

Based on digital animation stage education randomized controlled trial participants were randomly assigned to digital animation intervention group and routine treatment group. In addition to conventional treatment, patients in the intervention group received multi-period digital animation education. Firstly, the animation education for patients during hospitalization mainly focused on the pathogenesis and harm of AF. Secondly, animation education for patients after hospitalization focused on the treatment and prognosis of AF. Thirdly, animation education related to preoperative preparation and precautions was carried out before surgery. Then, the digital animation education for patients after surgery mainly focused on postoperative nursing and postoperative precautions. Finally, animation education about post-discharge medicine and life advice was carried out for patients

before discharge.Patients in the control group only received conventional treatment. The routine treatment of patients in the intervention group and the control group was strictly carried out in accordance with the guidelines for AF.

Participate in the intervention group and control group patients were age, gender, history, height, weight, smoking, alcohol and other baseline data collection, in addition, in intervention group and control group of patients admitted to hospital at the same time completed the self-rating anxiety scale [SAS]; self-rating depression scale [SDS]; the quality of life in patients with atrial fibrillation [AF-Qol-18]; medication adherence report scale [MARS5]. The patients were followed up by telephone at 3 months after discharge, and the SAS, SDS, AFQ-18 and MARS5 were collected again. At the same time, the patients were followed up for 3 months after discharge, including daily exercise, smoking, drinking, staying up late, and changes in dietary habits.

Study Duration

Participants were required to receive the intervention throughout their hospital stay and to be followed up at 3 months after discharge.

Study Population

The randomized controlled trial included 208 AF patients who underwent AFCA in the Department of Cardiology at Renmin Hospital of Wuhan University from January 2022 to August 2023. Main inclusion criteria were as follows: (1) Age between 18 and 90 years old; (2) Confirmed diagnosis of AF by surface electrocardiogram or ambulatory electrocardiogram examination; (3) AF patients who underwent successful RFA during hospitalization at Wuhan University People's Hospital. The diagnosis and treatment criteria for AF strictly followed the guidelines for AF management. Participants who were unable to comply with the study (as determined by clinical researchers) or who had a history of cardiac surgery (including percutaneous coronary intervention, coronary artery bypass grafting (CABG), or heart transplantation) were excluded.

Inclusion and exclusion criteria.

Inclusion criteria
Age between 18 and 90 years old
Confirmed diagnosis of AF by surface electrocardiogram or ambulatory electrocardiogram examination
AF patients who underwent successful AFCA during hospitalization at Wuhan University People’s Hospital
**Exclusion criteria**
Previous cardiac surgery
Out of age
Refusal to sign consent form
Mental abnormality
Not suitable for participation

Early termination of the study

This study may be temporarily suspended or terminated early if there is good and reasonable cause for such termination.

Suspended or terminated criteria
Determination of unexpected, significant or unacceptable risks to participants
Identify patients who may be at risk for an Serious Adverse Events(SAE)
Inadequate compliance with protocol requirements by the investigator

Interim analyses

No interim analyses were planned or conducted.

Screening, Randomization, and Follow-up Flowchart

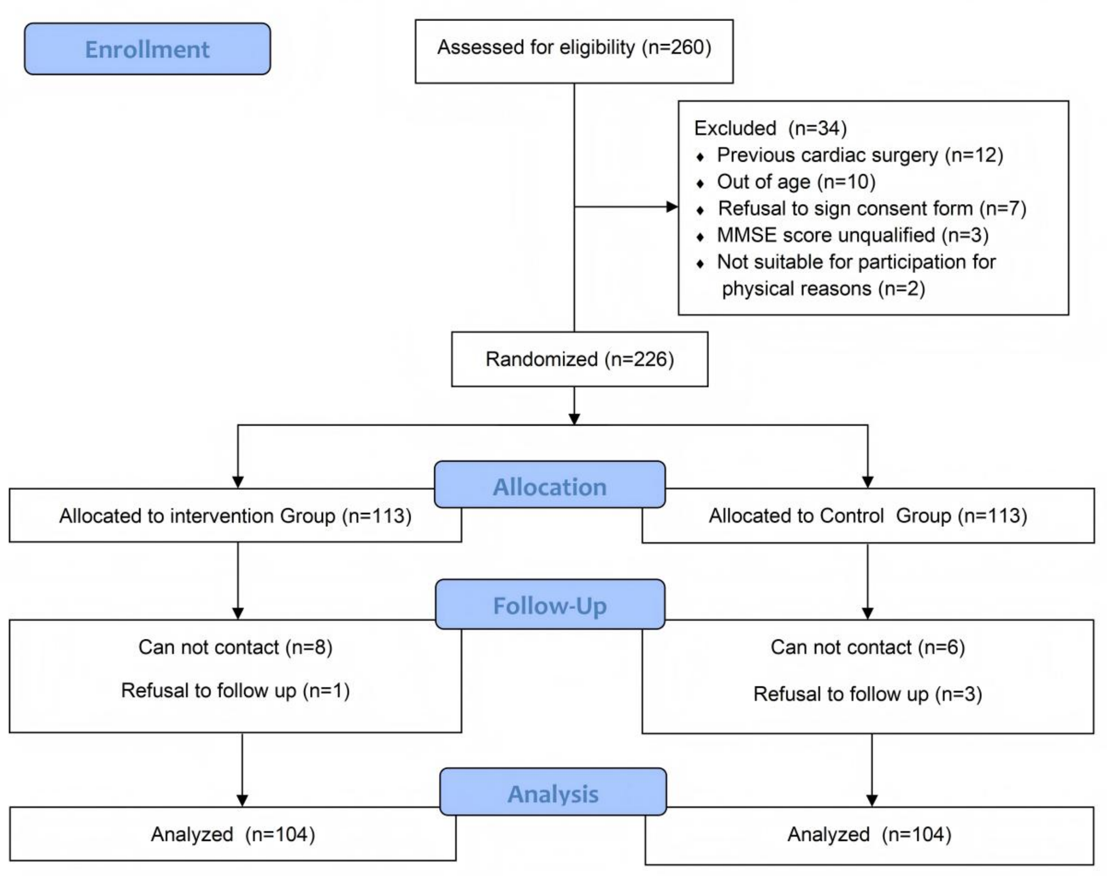

Study Procedures

Intervention Strategy

The main purpose of multi-stage digital animation education is to carry out popular science intervention for patients with AF based on the Chinese real estate management guidelines, improve patients' cognition of AF disease, help patients relieve their bad emotions during hospitalization, change the bad living habits of patients and even healthy people, and prevent and reduce the risk factors inducing AF. Intervention will continue in all patients during hospitalization, intervention content mainly includes: 1) monitoring the quality of life of AF patients; 2) monitoring in patients with AF patients hospitalization anxiety; 3) monitor the depression of patients during hospitalization; 4) to improve patients' medication compliance.

So far, clinical treatment revolves around the pathophysiology of the patient's disease, and often ignores the patient's psychological problems, including but not limited to anxiety, depression and other negative emotions. Patients with anxiety, depression, bad mood and mental illness, unknown, worry about prognosis and

surgical risk for the disease had close relations, etc. To assess patients with anxiety, depression, bad mood, you first need an objective solution to uniform standards.

Establish a popular science mode based on digital animation. Digital animation is a new, humorous, portable and easy-to-understand media video. Popular science education has been shown to improve the cognition to the disease, patients improve patient adherence and assist patients to establish a good way of life, reduce disease risk factors. Ever more popular science education depends on form of text, images, content is relatively dry, less patient acceptance and understand the degree of difference. The popular science mode based on digital animation is vivid, vivid, humorous and interesting, which is easy for patients to understand and deepen their learning degree of popular science content.

Based on digital animation science auxiliary medical personnel to complete the doctor-patient communication during the patient in the hospital. Doctor-patient communication is an important part of medical decision-making, which affects the prognosis of patients and runs throughout their hospitalization. Efficient and warm doctor-patient communication can effectively reduce medical conflicts and improve patient satisfaction, so as to quickly implement treatment in the occurrence and development of patients' diseases. Based on digital animation mode of doctor-patient communication more efficient, help patients quickly realize his illness, actively cooperate with treatment.

We developed a series of four animation videos for patients, each tailored to different stages of their care: upon admission, preoperatively, postoperatively, and at discharge. The production of these animations was funded by the Department of Cardiology at Renmin Hospital of Wuhan University, which holds the intellectual property rights. Currently, public access to these animations is restricted. However, we recognize their potential value to a broader audience and are considering further optimizations to facilitate wider dissemination in the future.

Control group

Patients in the conventional treatment group were required to complete the SAS,SDS,AFQ-18 and MARS5 scales during hospitalization and 3 months after

discharge, and received normal doctor-nurse communication and standardized treatment during hospitalization. Standardized treatment was carried out in strict accordance with the Chinese guidelines for the management of AF. In addition, all AF patients were provided with lifestyle advice to reduce the risk factors of AF through oral education by physicians.

Randomization

The study employed an online randomization tool to allocate 208 patients undergoing radio-frequency ablation into either the digital animation intervention group or the usual care group with equal probability. Stratification based on gender and age (>60 or ≤60 years) was conducted to ensure balance in baseline characteristics between the two groups.

Blinding

Neither the participants, treating clinicians, nor the researchers were blinded.

Study Outcome

Two groups at baseline and 3 months after discharge to check twice. Evaluation index including 3 months after the quality of life score (self-rating anxiety scale [SAS], self-rating depression scale [SDS], the quality of life in patients with AF [AFQ-18] and medication Adherence Report Scale [MARS5]).

All patients with AF included in the study, regardless of whether they were assigned to the intervention group or the control group, the above experimental results were only collected at admission and the 3-month follow-up after discharge. There was no follow-up and no telephone intervention during the 3-month period after discharge.

Statistical Method:

Statistical analyses were conducted using SPSS 23.0 and R software version 4.2.2. For data that followed a normal distribution, means ± standard deviations (±SD) were used for representation, and comparisons between the two groups were made using the independent samples t-test. For data that did not follow a normal distribution,

medians and interquartile ranges (IQR) were used, with the Mann-Whitney U test applied to analyze differences between groups. Categorical data were expressed in numbers and percentages, and compared using the chi-square test or Fisher's exact test as appropriate. To assess the effectiveness of the intervention, differences between the two groups were calculated by subtracting the change from baseline to 3 months in the control group from that in the intervention group. An independent t-test was then conducted to determine differences between groups before and after the intervention. A p-value of less than 0.05 was considered statistically significant.

Sample Size Consideration

The sample size estimation was based on a comparison of two independent proportions, with a Type I error rate (α) set at 2.5%, an intra-cluster correlation coefficient (ICC) of 0.01, and an anticipated retention rate of 80%. Our objective is to detect a significant absolute difference of 20% in clinical outcome between the intervention and standard treatment groups with a statistical power of 90% at a follow-up period of 3 months. Based on our calculations, it is determined that a minimum sample size of 80 assessable patients per group is required to achieve this desired level of statistical power.

Informed Consent

Multi-stage digital animation education research trials of recruiting, screening, and into the group of participants in wuhan university people's hospital. We through the electronic system of the hospital and outpatient clinic to determine potential qualified patients, and arrange the next recruitment consultancy. We consulted all potential patients who met the major inclusion criteria on-site at the time of admission. In the process of participants selected, willing to participate in trials of patients will be required to allow to input the information into the database, for the next step screening of patients. Patients is expected at the beginning of the screening visit to conduct a comprehensive screening, to evaluate the qualification test. Patients who pass the screening visit will be considered eligible to enter the informed consent process. During this process, we will provide eligible patients with a detailed verbal

description of the study, including its risks, potential benefits, and requirements. We will also provide a paper informed consent form for eligible patients to read, and patients will have ample time to read and reflect on participation. If required, the individual will be given additional time to consider participation, including rearranging the screening visit. Before get written consent, everyone can ask questions, until individual decision making. When ready, participants will be asked to sign the consent form. Then, we will collect the data to provide informed consent of patients, including from the eligible patients' electronic health records and research the information of the program.

Appendix. Informed Consent

Informed Consent

Title: Efficacy of a Digital Animation-based Multistage Education in Patients with AF Catheter ablation : A Randomized Clinical Trial.

Informed Consent • Informed Notice page

Dear participant,

We would like to extend an invitation for you to take part in Efficacy of a Digital Animation-based Multistage Education in Patients with AF Catheter ablation. This study has undergone a thorough review and has been approved by the Ethics Committee of Renmin Hospital of Wuhan University.

Before making a decision about participating in this clinical study, we kindly ask you to carefully read the following information. This will help you understand the study's purpose, duration, requirements, and potential benefits, risks, and discomforts associated with participation. Please note that your participation is voluntary, and you have the right to choose whether to participate or not.

You may discuss this study and the information provided with your family, friends, doctor, or other trusted individuals. The study doctor will explain this information to you, and if you have any questions, they will be happy to answer them.

If you decide to participate, you will be asked to sign an informed consent form (at the end of this document) before any study-related procedures are performed.

Introduction

Atrial Fibrillation (AF) is the most common cardiac arrhythmia, significantly increasing the risk of mortality, stroke, heart failure, and dementia, thereby severely impacting patients' quality of life. It is estimated that by 2050, at least seventy-two million Asians will be diagnosed with AF. The primary goals of treating AF include symptom improvement, heart rate and rhythm control, and stroke risk reduction. AF Catheter ablation (AFCA) is currently the mainstay treatment for AF, aiming to eliminate cardiac aberrant pacemaker sites that trigger AF, and has become a pivotal strategy for rhythm control. However, the long-term prognosis for some patients remains suboptimal.

Studies indicate that negative emotions, alcohol consumption, smoking, obesity, and sleep disorders, among other high-risk factors, can expedite AF progression and prognosis, potentially directly impacting the efficacy of AFCA and postoperative recovery. Additionally, anticoagulation therapy significantly reduces stroke risk in AF patients, emerging as one of the crucial factors affecting treatment efficacy. Therefore, alongside enhancing the safety and efficacy of catheter ablation for AF, strategies focusing on risk factor management and medication adherence to improve ablation outcomes are deemed essential in AF patient management.

Popular science interventions such as animations and comics represent a non-invasive, entertaining emerging approach to disease intervention, employed to ameliorate patients' emotions, sleep, lifestyle, and disease prognosis. Through vivid and engaging animation content, patients can vividly comprehend various aspects of AF, including etiology, pathology, surgical procedures, and complications, thereby enhancing patients' interest and engagement in health management. A recent study suggests that employing animation education can enhance AF patients' understanding of the disease and increase satisfaction with clinical care.In this prospective, randomized, controlled clinical trial, we aim to evaluate the efficacy of multi-stage education based on digital animation for AF patients undergoing catheter ablation.

Research Purpose:

Primary Purpose: To assess the improvement in quality of life among patients with AF three months post-discharge.

Secondary Purpose: To evaluate the changes in anxiety, depression, and medication adherence among AF patients three months after discharge.

Inclusion and exclusion criteria.

**Inclusion criteria**
Age between 18 and 90 years old
Confirmed diagnosis of AF by surface electrocardiogram or ambulatory electrocardiogram examination
AF patients who underwent successful AFCA during hospitalization at Wuhan University People's Hospital
**Exclusion criteria**
Medical history of percutaneous coronary intervention or coronary artery bypass grafting
Out of age
Refusal to sign consent form
Mental abnormality
Not suitable for participation

How many people will participate in this study?

The plan is to recruit 208 subjects in this study at our institution.

Study Procedure

(1) Prior to your inclusion in the study, the research team will collect the following information and examination results to determine your eligibility for participation:

Medical History: The study physician will inquire about any current or past medical conditions.

Demographic Information: The study physician will gather personal information, including your date of birth and ethnic background.

Laboratory Results: Blood biochemistry and complete blood count will be assessed.

Height and Weight: Research personnel will measure your height and weight.

Vital Signs: The study physician will record your blood pressure, heart rate, temperature, and respiratory rate.

Holter Monitor Results: Dynamic electrocardiogram results will be obtained.

Echocardiogram Results: Results from echocardiographic examinations will be collected.

(2) If the above information and results meet the eligibility criteria, you will be confirmed for inclusion in the study. You will then be randomly assigned to either the control group or the video intervention group, with a treatment duration of one week (including experimental treatment and random group assignment). During this treatment period, researchers will observe the actual effects of the video intervention or control intervention. To ensure accurate recording and assessment of the video’s effects, your cooperation is required in completing the following assessments:

•self-rating anxiety scale [SAS]

•self-rating depression scale [SDS]

•the quality of life in patients with atrial fibrillation [AF-Qol-18]

•medication adherence report scale [MARS5]

(3) Upon completing all treatments, researchers will conduct a follow-up via telephone or request that you return for a follow-up visit 12 weeks after treatment completion. During this period, the following assessments will be conducted:

•self-rating anxiety scale [SAS]

•self-rating depression scale [SDS]

•the quality of life in patients with Atrial Fibrillation [AF-Qol-18]

•medication adherence report scale [MARS5]

(4) Upon completion of all follow-ups, you will be considered to have finished the study.

Please

How long will this study last?

This clinical trial will be conducted over a period of 3 months, during which you will be required to attend Telephone follow-up visits at 3 months. You have the right

to withdraw from the study at any time without fear of discrimination or retaliation, and your decision to withdraw will not affect your medical treatment or rights. Your clinician or researcher may also suspend your participation in the study at any time if it is deemed to be in your best interest (the reason for possible termination of the trial will be explained to you). If you choose to participate in this study, we kindly request that you commit to completing the entire research process. In the event that you withdraw from the study for any reason, a relevant examination may be conducted to ensure your safety.

1. Risks and/or discomforts of participating in this study

There are no risks associated with participating in this study. However, there may be information security risks. We will do our best to protect the information you provide from being disclosed. Some of the questions we ask you in this study may make you feel uncomfortable, and you have the right to refuse to answer such questions. Additionally, you can take a break at any time during the study. At any point during the study, you can choose to withdraw from the study.

2. Benefits of participating in the study

If you agree to participate in this study, you may potentially receive direct medical benefits. Specifically, this study aims to enhance your understanding of AF. However, we cannot guarantee this outcome. We hope that the information we obtain from your participation in this study will help provide more information for the diagnosis and treatment of AF in the future.

3. Alternative treatment options if not participating in the study

This study will not provide any other treatment options. Your diagnosis and treatment will be determined by the research doctor based on your condition, and you can continue with your regular treatment plan.

The use of research results and confidentiality of personal information: In this study, your personal information will be collected for statistical and analytical purposes. You will have the opportunity to learn about the research results. You can ask your research doctor for the results and ask for an explanation. The results of this study may also be published in journals or presented at conferences, but they will not

contain any information that could identify you.

To ensure privacy, records or samples published for research purposes will not include your name or any other identifying information. Instead, your information will only be identified by a code. Only the research doctor and authorized personnel can link this code to your name through a list, which will be securely stored at the research center.

In order to ensure that the research is conducted in accordance with regulations, the applicant, ethics review committee, and government regulatory agencies may access your information when necessary. They are bound by confidentiality obligations and will not violate your privacy.

You have the right to control the use and disclosure of your personal information. You can request to view your medical information at any time, as permitted by national law. You have the right.

Research-related updates

During the course of the study, if there are any changes to the study protocol or application, your research doctor will immediately inform you and discuss with you whether you wish to continue participating in the study. If you decide not to continue, your medical treatment and rights will not be affected. If you choose to remain in the study, your research doctor may ask you to sign a new informed consent form.

Study costs, compensation, and damages

If you participate in this study, you will not be required to pay any additional fees. You will only be responsible for the costs associated with the relevant medical examinations for your underlying condition. You will not receive any financial compensation for your participation in the study. However, during the follow-up process, you will receive health guidance from our professional medical staff. If you suffer any harm as a result of participating in the study, you will receive professional treatment provided by the department of cardiology of Renmin Hospital of Wuhan University, and will be compensated in accordance with the law.

Rights and Responsibilities of Participants

1. Rights

Throughout the entire research process, your participation is voluntary. If you decide not to participate in this study, it will not affect any other treatment you may receive. If you choose to participate, you will be asked to sign this informed consent form. You have the right to withdraw from the study at any time without discrimination or unfair treatment, and your medical treatment and rights will not be affected.

2. Responsibilities

As a participant in this study, please abide by the following agreements:

• Return to the hospital for scheduled visits on time.

• You can inform your research doctor at any time if you wish to terminate the study.

• Provide truthful information about your medical history and current physical condition.

• Follow the instructions of the research staff.

• Inform the research doctor of any discomfort you experience during the study.

• Any experimental treatment may pose a risk to you or your fetus, so you and your partner should avoid any activities that may lead to pregnancy during the study. If you become pregnant during the study, please inform your research doctor immediately.

3. Contact Information

If you have any questions related to this study, please contact the researcher at 15540963659.

If you have any questions regarding your rights and interests, or if you wish to report any difficulties, dissatisfaction, or concerns during your participation in this study, or if you wish to provide feedback or suggestions related to this study, please contact the Ethics Committee of Renmin Hospital of Wuhan University at 027-88041911-81353.

Informed Consent Form • Consent Signature Page

Participant Declaration:

I have been given information about the study's background, purpose, methodology, potential risks, and benefits. I have had ample time and opportunity to ask questions, and I am content with the responses I have received. Additionally, I have been made aware of who to contact if I have any inquiries, concerns, recommendations, or would like to offer additional information or support for the study.

I acknowledge that my participation in this study is entirely voluntary, and I affirm that I have been given ample time to carefully consider and willingly consent to take part. I retain the right to withdraw from the study at any point without fear of any negative consequences or repercussions on my medical treatment or personal rights. Additionally, I have been assured that the researchers have not employed any deceitful tactics, coercion, or undue pressure to compel my participation in the study.

I acknowledge that in the event of my condition deteriorating, or if I encounter severe adverse reactions, or if my research doctor deems that my continued participation in the study is not in my best interest, he/she may withdraw me from the study. Additionally, the sponsor or regulatory agency may terminate the study during the research period without my consent. In such an event, my doctor will inform me promptly, and my research doctor will discuss alternative options with me.

I have carefully reviewed and understood the contents of this informed consent form, and I willingly consent to participate in this study. I acknowledge that I will be provided with a copy of the original informed consent form, which will include both my and the researcher's signature and the date of signing.

Participant Signature: Date:

Contact Phone Number:

Legal Representative Signature: Date:

Contact Phone Number:

(Note: If the participant has no legal capacity or limited legal capacity, such as inclusion of vulnerable groups with mental disorders/unconsciousness, the legal representative needs to sign at the following legal representative signature)

Fair Witness Signature: Date:

Contact Phone Number:

(Note: Only when it is possible to include participants with decision-making capacity but unable to read the text, such as illiteracy, visual impairment, a fair witness signature is required. The researcher should keep video materials as proof of informed consent when the witness is informed.)

Researcher Declaration:

I have accurately informed the participant of this document, and he/she has read this informed consent form accurately and confirmed that the participant had the opportunity to ask questions and voluntarily agreed. I have given him/her a signed original of the informed consent form.

Researcher Signature: Date:

Contact Phone Number:

Reference

[1] ANDRADE J G, MACLE L, NATTEL S, et al. Contemporary Atrial Fibrillation

Management: A Comparison of the Current AHA/ACC/HRS, CCS, and ESC Guidelines[J]. Can J Cardiol, 2017,33(8): 965-976.

[2] CHIANG C E, WANG K L, LIP G Y. Stroke prevention in atrial fibrillation: an Asian perspective[J]. Thromb Haemost, 2014,111(5): 789-797.

[3] THRALL G, LANE D, CARROLL D, et al. Quality of life in patients with atrial fibrillation: a systematic review[J]. Am J Med, 2006,119(5): 441-448.

[4] THRALL G, LIP G Y, CARROLL D, et al. Depression, anxiety, and quality of life in patients with atrial fibrillation[J]. Chest, 2007,132(4): 1259-1264.

[5] CALKINS H. Catheter ablation to maintain sinus rhythm[J]. Circulation, 2012,125(11): 1439-1445.

[6] WALD D S, CASEY-GILLMAN O, COMER K, et al. Animation-supported consent for urgent angiography and angioplasty: a service improvement initiative[J]. Heart, 2020,106(22): 1747-1751.

[7] BRAND A, GAO L, HAMANN A, et al. Medical Graphic Narratives to Improve Patient Comprehension and Periprocedural Anxiety Before Coronary Angiography and Percutaneous Coronary Intervention: A Randomized Trial[J]. Ann Intern Med, 2019,170(8): 579-581.
