# Supplementary material for: The Effectiveness of Digital Animation–Based Multistage Education for Patients With Atrial Fibrillation Catheter Ablation: Randomized Clinical Trial
Source: J Med Internet Res. 2025 Mar 11;27:e65685. doi: 10.2196/65685 (PMC11937711; doi:10.2196/65685)
Supplement: Multimedia Appendix 3 [file jmir_v27i1e65685_app3.pdf]

eTable 1. Mean Quality-of-life total score (Comparison within groups).

Scale,mean (SD)	Group	Baseline	Three Months	t	P
**AF-Qol-18 score**	Intervention	38.02±6.52	47.77±5.74	27.844	<.001
	Control	36.97±7.00	45.31±5.71	22.269	<.001
**MARS-5 score**	Intervention	17.04±3.03	20.13±2.12	12.038	<.001
	Control	17.14±3.01	18.47±2.79	6.199	<.001
**SAS score**	Intervention	52.82±8.08	45.39±6.13	18.771	<.001
	Control	51.83±7.74	47.31±5.87	15.540	<.001
**SDS score**	Intervention	54.12±6.13	45.47±5.94	22.638	<.001
	Control	52.78±5.21	45.37±6.18	15.360	<.001

eTable 2. Mean Quality-of-life total score (comparison among groups).

**Scale**,mean (SD)	**Intervention（n=104）**	**Control（n=104）**	**t**	**P**
**AF-Qol-18 score**				
Baseline	38.02±6.52	36.97±7.00	1.117	.265
Three Months	47.77±5.74	45.31±5.71	3.102	.002
**MARS-5 score**				
Baseline	17.04±3.03	17.14±3.01	0.252	.801
Three Months	20.13±2.12	18.47±2.79	4.812	<.001
**SAS score**				
Baseline	52.82±8.09	51.83±7.74	0.902	.368
Three Months	45.39±6.13	47.31±5.87	2.309	.022
**SDS score**				
Baseline	54.12±6.13	52.78±5.21	1.694	.092
Three Months	45.47±5.94	45.37±6.18	0.126	.900

eTable 3. Quality-of-life total score reduction 3 months from baseline.

**Scale**,mean(95% CI)	Intervention（n=104）		Control（n=104）		Difference change from baseline	P
	Mean	Score change	Mean	Score change		
**AF-Qol-18 score**						
Baseline	38.02（36.75-39.29）		36.9（35.61-38.33）			
Three Months	47.77（46.65-48.89）	9.75（10.44- 9.06）	45.3（44.20-46.42）	8.34（9.08- 7.59）	1.41 (2.42 to 0.40)	.006
**MARS-5 score**						
Baseline	17.04（16.45-17.63）		17.1（16.56-17.73）			
Three Months	20.13（19.71-20.54）	3.09（3.60- 2.58）	18.4（17.93-19.01）	1.33（-1.75- 0.90）	1.76 (2.42 to 1.10)	<.001
**SAS score**						
Baseline	52.82（51.24-54.39）		51.8（50.32-53.33）			
Three Months	45.39（44.19-46.58）	-7.43（-6.65--8.22）	47.3（46.17-48.15）	-4.52（-3.94--5.10）	-2.91（-3.88 to -1.95）	<.001
**SDS score**						
Baseline	54.12（52.92-55.31）		52.7（51.77-53.79）			
Three Months	45.47（44.32-46.63）	-8.64（-7.89--9.40）	45.3（44.16-46.57）	-7.41（-6.46--8.37）	-1.23（-0.02 to -2.44）	.047

eTable 4. Heart rate and atrial arrhythmia events at 3 months follow-up.

Outcome	Intervention（n=104）	Control（n=104）	t/Z/χ^2	P
**Heart Rate**	70.08 ± 9.62	70.85 ± 9.46	1.147	0.253
**Atrial Arrhythmia Events**[a], n (%)	9（8.65）	13（12.5）	0.457	0.499
